# Coherent control of the silicon-vacancy spin in diamond

**DOI:** 10.1038/ncomms15579

**Published:** 2017-05-30

**Authors:** Benjamin Pingault, David-Dominik Jarausch, Christian Hepp, Lina Klintberg, Jonas N. Becker, Matthew Markham, Christoph Becher, Mete Atatüre

**Affiliations:** 1 Cavendish Laboratory, University of Cambridge, JJ Thomson Avenue, Cambridge CB3 0HE, UK; 2 Fachrichtung 7.2 (Experimentalphysik), Universität des Saarlandes, Campus E2.6, 66123 Saarbrücken, Germany; 3 Element Six Ltd., Global Innovation Centre, Fermi Avenue, Harwell Oxford, Didcot OX11 0QR, UK

## Abstract

Spin impurities in diamond have emerged as a promising building block in a wide range of solid-state-based quantum technologies. The negatively charged silicon-vacancy centre combines the advantages of its high-quality photonic properties with a ground-state electronic spin, which can be read out optically. However, for this spin to be operational as a quantum bit, full quantum control is essential. Here we report the measurement of optically detected magnetic resonance and the demonstration of coherent control of a single silicon-vacancy centre spin with a microwave field. Using Ramsey interferometry, we directly measure a spin coherence time, *T*_2_*, of 115±9 ns at 3.6 K. The temperature dependence of coherence times indicates that dephasing and decay of the spin arise from single-phonon-mediated excitation between orbital branches of the ground state. Our results enable the silicon-vacancy centre spin to become a controllable resource to establish spin-photon quantum interfaces.

## Abstract (web summary)

Silicon vacancy centres in diamond have favourable optical properties for use in quantum information processing. Here, the authors demonstrate coherent control of silicon vacancy spins, a prerequisite for the implementation of quantum computing operations.

Spin-free materials, such as diamond and silicon, are ideal hosts to investigate and control the dynamics of spin-carrying defects. Among such systems, the nitrogen-vacancy (NV) centre in diamond has attracted great interest for electromagnetic field sensing^1^,^2^,^3^,^4^, bio-labelling^5^,^6^ and quantum information processing^7^,^8^,^9^,^10^,^11^. However, the NV centre only emits around 4% of its fluorescence into the zero-phonon line, limiting its use for quantum information processing in the absence of elaborate photonic structures such as photonic cavities^12^. In contrast, the negatively charged silicon-vacancy centre in diamond (SiV^−^) emits around 80% of its photons into the zero-phonon line^13^, with optical properties characterized by spectral stability and narrow inhomogeneous distribution in bulk diamond^14^. This makes the SiV^−^ an ideal building block for a distributed quantum network^15^,^16^. Furthermore, the SiV^−^ offers two possible realizations of a quantum bit: one consists in using the two readily available orbital branches of the ground state, split by 50 GHz (ref. 17). The other takes advantage of the SiV^−^ ground-state spin *S*=1/2, optical signatures of which have previously been reported^18^ and which promises longer coherence times, as estimated through coherent population trapping^19^,^20^. Using the spin as a quantum bit allows tuning of the resonance frequency with a magnetic field in the few GHz range, routinely used for NV centres. Achieving coherent control of this spin is a fundamental step for the implementation of spin-based quantum computing algorithms. Using microwave control provides more flexibility in the orientation of the magnetic field than all-optical control, which requires lambda-type transitions to be allowed. Furthermore, the capacity to address the spin state of the SiV^−^ with microwave pulses is crucial to use the SiV^−^ as an interface between optical and microwave photons for hybrid quantum computing^21^. Similar efforts are also undertaken with other colour centres in diamond^22^.

Here we report the realization of coherent control of the silicon-vacancy centre electronic spin. We perform optically detected magnetic resonance (ODMR) by tuning the frequency of a microwave field into resonance with the Zeeman splitting between two electronic spin states of a single SiV^−^ centre. This allows us to perform spectroscopy of the magnetic dipolar transitions between ground-state levels of opposite electronic spin. By fixing the frequency of the microwave field in resonance with one of those transitions and varying the duration of the pulse, we coherently drive the SiV^−^ electronic spin. We then perform Ramsey interferometry and measure a spin dephasing time of 115±9 ns at 3.6 K. The temperature dependence of the spin coherence times reveals that spin dephasing and population decay are dominated by single-phonon excitation to the upper orbital branch of the ground state.

## Results

### Spin initialization and read-out

**Figure 1:**
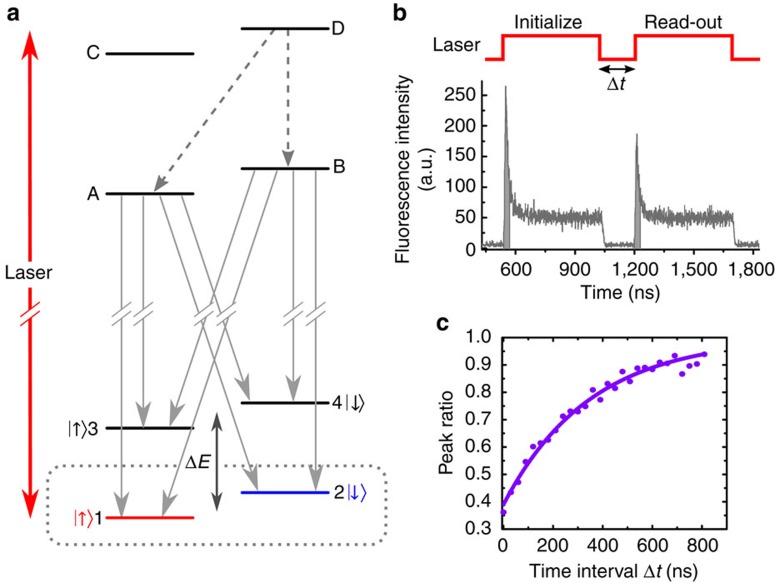
Spin initialization and read-out. (**a**) Energy level scheme for a SiV^−^ under magnetic field. Excited-state levels are labelled from A to D. States labelled 3 and 4 correspond to the upper orbital branch of the ground state. The dotted grey box highlights the Zeeman-split lower orbital branch, on which we focus in this work and where state 1 (in red) corresponds to a spin-up state and state 2 (in blue), to a spin-down state. The ground state upper orbital branch lies approximately at Δ*E*=50 GHz above the lower one. A tunable laser at a wavelength of ∼737 nm (red double arrow) drives transition D1 resonantly. Grey dashed arrows show fast decay paths from excited state D to excited states A and B. Solid grey arrows identify the measured fluorescent transitions. (**b**) A first 500 ns-long optical pulse resonant with transition D1 causes optical pumping into the spin-down state, as evidenced by the exponential decay of the fluorescence. The height of the leading edge of the fluorescence due to the second 500 ns-long pulse indicates the recovery of the spin-up population, and thus acts as a read-out. The dark grey areas under the leading edges of the initialization and read-out pulses correspond to a duration of 30 ns during which the signal is integrated to calculate the peak ratio. (**c**) Recovery of the ratio between the leading edge of the read-out pulse with respect to the initialization pulse as a function of the time interval between the pulses at 3.5 K (purple dots). The purple curve is an exponential fit with 1/*e* value *T*_1,spin_= 350±11 ns.

The SiV^−^ centre is composed of a silicon atom replacing two neighbouring carbon atoms in the diamond lattice (Supplementary Note 1). Its energy levels are characterized by orbitally split ground and excited states (Fig. 1a). We study single SiV^−^ centres in bulk diamond, created by implantation of isotopically purified ^29^Si followed by annealing (see the ‘Methods' section). An external magnetic field lifts the degeneracy of the electronic spin *S*=1/2, resulting in the energy level diagram shown in Fig. 1a. In our experiment, this magnetic field is applied at an angle of 109.5°±1° with respect to the SiV^−^ symmetry axis, which dictates that all transitions between ground and excited states are optically allowed^19^. We use an optical pulse from a diode laser tuned to resonance with transition D1 (between ground-state level 1 and excited-state level D, red double arrow in Fig. 1a) to pump the SiV^−^ optically into the spin-down ground state. Fluorescence from the SiV^−^ is collected on the remaining transitions (solid grey arrows in Fig. 1a) following thermalization among excited states (dashed grey arrows in Fig. 1a). The decrease of fluorescence during the laser pulse (Fig. 1b) allows to extract an initialization fidelity of about 85% through a master equation model (Supplementary Note 5). We perform the spin read-out analogously by measuring the recovery of the leading edge of the fluorescence generated by a second optical pulse resonant with the same transition. The spin-state measurement corresponds to the peak ratio between the integrated areas under the leading edge of the read-out pulse and that of the initialization pulse. By varying the time delay between the two pulses, we extract from the exponential recovery a spin relaxation time *T*_1,spin_=350±11 ns (Fig. 1c) at 3.5 K, setting an upper time bound for subsequent driving of the SiV^−^ spin in this configuration.

### Optically detected magnetic resonance

**Figure 2:**
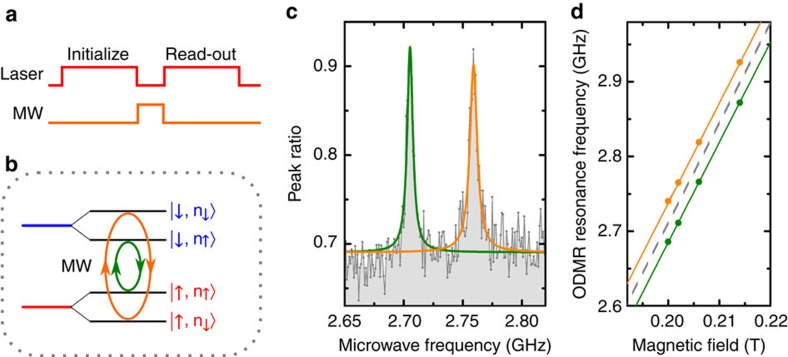
Optically detected magnetic resonance. (**a**) ODMR pulse scheme. A 140 ns microwave pulse is applied during the 160 ns interval between optical initialization and read-out pulses at a measured temperature of 4.3 K. (**b**) Close-up on the Zeeman-split lower branch of the ground state. The hyperfine interaction with the ^29^Si nuclear spin ½ causes a splitting of each electronic spin state. An applied microwave can flip the electronic spin, leaving the nuclear spin unchanged, resulting in two possible resonance frequencies (orange and green circular arrows). (**c**) ODMR spectrum: the peak ratio is plotted as a function of the microwave frequency. The two microwave resonances appear as two peaks, each fitted with a Lorentzian function (colours correspond to those of **b**) and separated by 53.7±0.3 MHz (error from Lorentzian fit). The ODMR contrast calculated as the ratio between the peak height and the baseline is about 33%. (**d**) ODMR resonance frequencies as a function of external magnetic field (orange and green dots). Error bars are smaller than dots and correspond to the s.d. of ∼0.2 MHz on the central frequency of the ODMR peak Lorentzian fits. The solid curves are fits based on the model developed in ref. 23 expanded to include the nuclear spin. Orange and green curves correspond to the transitions in **b**, the two overlapping dashed grey curves correspond to the transitions where both electronic and nuclear spins are flipped.

To address the electronic spin of the SiV^−^, we apply a microwave pulse between the optical initialization and read-out pulses (Fig. 2a). Owing to the nuclear spin 1/2 of the ^29^Si isotope, each electronic spin state displays a hyperfine splitting, as depicted in Fig. 2b. The microwave field drives the electronic spin, while preserving the nuclear spin state, which results in two distinct microwave resonance frequencies (orange and green circular arrows in Fig. 2b). When on resonance with one of the two transitions, the microwave transfers population from the initialized spin state to the depleted one, resulting in a recovery of fluorescence during the read-out pulse and thus a peak in the ODMR spectrum with a contrast around 33% (Fig. 2c and Supplementary Note 3). We measure the frequencies of the two resonances at different values of the applied magnetic field, as shown in Fig. 2d. We fit the observed frequency shift using an effective Hamiltonian described in ref. 23, which includes the Jahn-Teller effect, spin–orbit coupling and electronic Zeeman effect, and accounts for the nuclear spin via the nuclear Zeeman effect and the hyperfine interaction (Supplementary Note 4). From the fit, we extract a value of *A*_||_=70±2 MHz for the hyperfine constant along the SiV^−^ axis, in agreement with previously reported experimental^20^,^24^ and theoretical^25^,^26^ values (Supplementary Note 4).

### Coherent control of the spin

**Figure 3:**
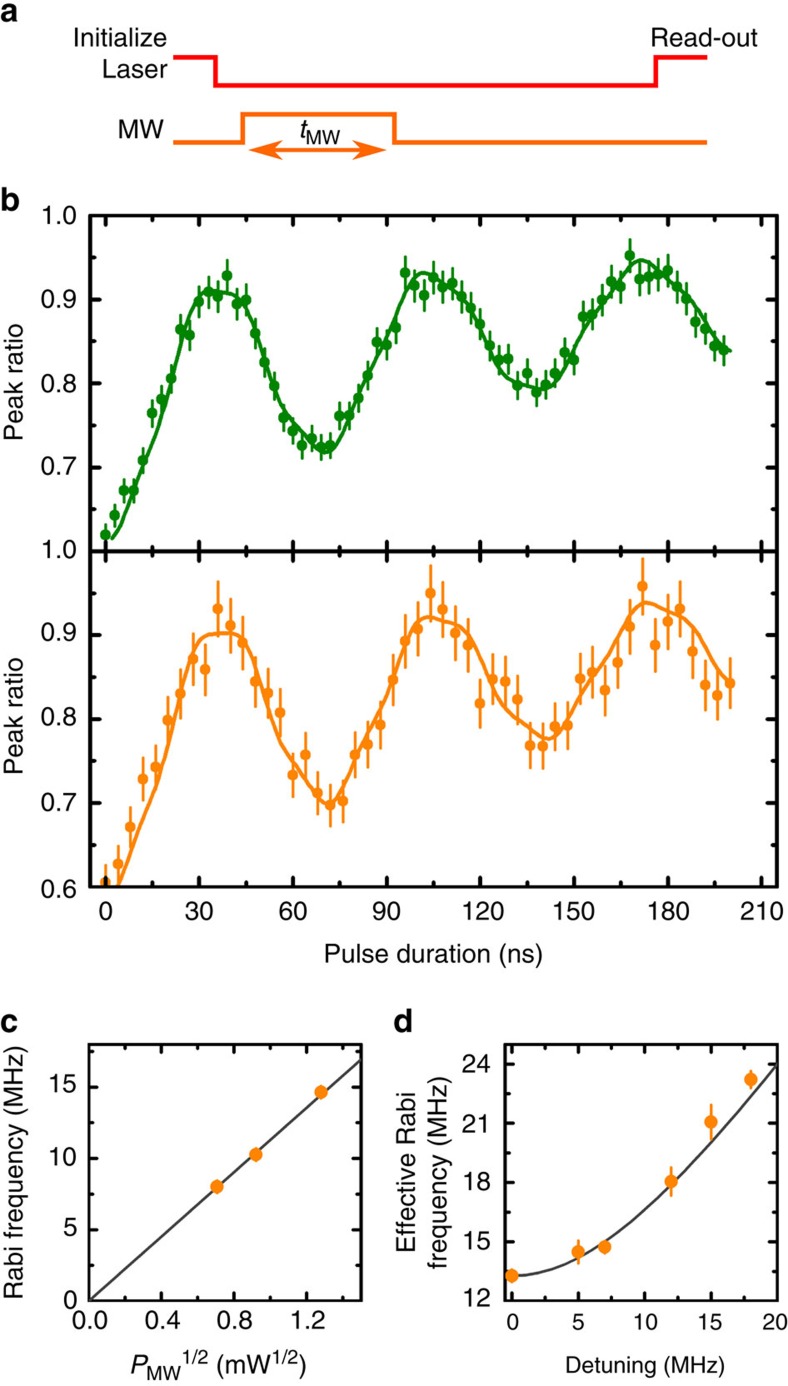
Rabi oscillations. (**a**) Microwave and optical pulses scheme: the frequency of the microwave is fixed at one of the microwave resonances and its duration *t*_MW_ is varied. The time interval between initialization and read-out pulses is kept constant at 210 ns, not to include the effect of the spin population decay into the measured signal. (**b**) Rabi oscillations between states 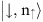 and 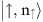 (green dots) and between states 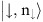 and 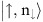 (orange dots) (corresponding to the green and orange circular arrows in Fig. 2b, respectively): the measured peak ratio between the leading edges of the read-out and initialization pulses oscillates as a function of the microwave pulse duration. The error bars correspond to the s.d. of the peak ratio (the difference in error bars between the two graphs is due to different integration times (see Methods)). The green (respectively orange) solid curve is a fit based on an eight-level master equation model (Supplementary Note 5). (**c**) Evolution of the Rabi frequency as a function of the square root of the input microwave power *P*_MW_, measured between states 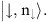 and 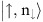. The error bars are smaller than dots. The grey line is a linear fit. (**d**) Effective Rabi frequency as a function of microwave frequency detuning, measured on transition 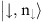 and 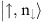 (orange dots). The error bars are the s.d. of the Rabi frequencies extracted from fits. The grey line is a fit of the form 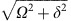, where *Ω* is the bare Rabi frequency and *δ* is the detuning.

Having determined the frequencies of the electron spin-flipping transitions, we fix the microwave frequency in resonance with one of them and vary the duration of the microwave pulse, as illustrated in Fig. 3a. Figure 3b shows the evolution of the read-out to initialization peak ratio with respect to the microwave pulse duration when the microwave pulse is resonant with the transition between states 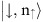 and 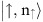 (green dots) and when it is resonant with the transition between states 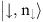 and 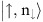 (orange dots). The signal displays Rabi oscillations, sign of the coherent driving of the electronic spin of the SiV^−^ (the nuclear spin orientation remaining unchanged). The upward drift of the oscillations arises from spin-preserving population transfers between the two orbital branches of the ground state. This is confirmed by an eight-level master equation model (Supplementary Note 5), used to reproduce the experimental curve (green and orange curves in Fig. 3b). This phenomenon highlights the importance of inter-branch dynamics at temperatures around a few kelvins. The detuned driving of the electron spin associated with the other nuclear spin orientation and of the transitions in the upper branch of the ground state (containing approximately one-third of the population at 4 K) is responsible for a decreased contrast of the oscillations and their slightly irregular shape (Supplementary Figs 7 and 8). The linear dependence of the Rabi frequency with the square root of the microwave power (Fig. 3c), as well as its variation with respect to detuning (Fig. 3d) confirms the coherent nature of those oscillations. The observed bare Rabi frequency of about 15 MHz is comparable to what is obtained with NV^−^ centres in similar conditions.

### Ramsey interferometry

**Figure 4:**
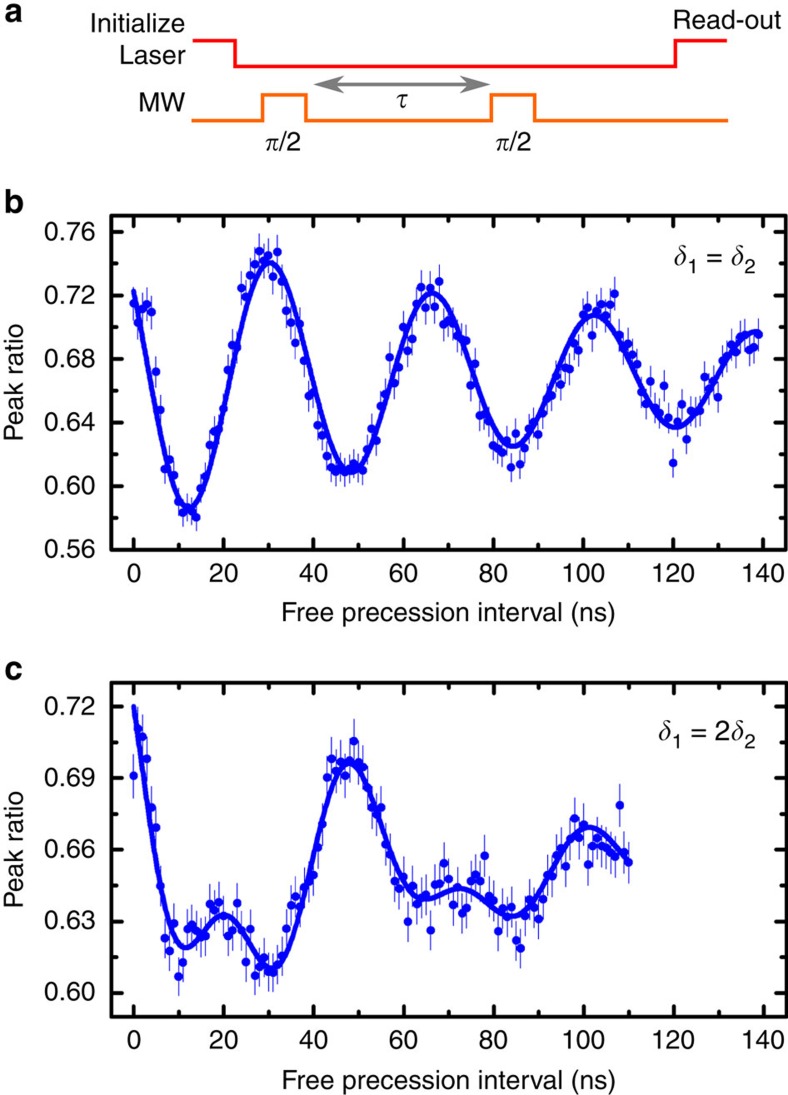
Ramsey interferometry. (**a**) Microwave and optical pulses protocol: two microwave *π*/2 pulses of 8 ns each are separated by a variable delay *τ*. The time interval between the initialization and read-out pulses is held constant at 160 ns, not to include the effect of the spin population decay into the signal. (**b**) Variation of the peak ratio as a function of the delay between the microwave pulses (blue dots) at 3.6 K (error bars correspond to the s.d. of the peak ratio). The microwave frequency is fixed at the average of the two resonance frequencies (the detuning *δ*_1_ from the lower frequency transition equals the detuning *δ*_2_ from the other). The blue solid curve is a fit of the form cos (2*π*·*τ*·*δ*) exp(−*τ*/*T*_2_*), where *δ* is the common detuning between the microwave frequency and the two resonances. We extract *T*_2_*=115±9 ns (the error corresponds to the s.d. on the fit parameter). (**c**) The duration of the microwave pulses is kept unchanged compared to **b**, but the frequency is tuned such that the detuning from one resonance is twice that with the other (*δ*_1_=36 MHz and *δ*_2_=18 MHz). The solid curve is a fit where the cosine function in **b** is replaced by the sum of two cosine functions, with frequencies corresponding to the respective detunings.

To measure the free induction decay time of the spin directly, we perform microwave Ramsey interferometry. We use a pulse sequence consisting of two *π*/2 microwave pulses separated by a variable delay (Fig. 4a). We fix the frequency of the microwave pulses such that in one case (Fig. 4b), the microwave frequency is in the middle of the two resonances, corresponding to a detuning of about 27 MHz from each of them, and in the other case (Fig. 4c), the detuning from one resonance equals twice that from the other (36 and 18 MHz, respectively). The measured peak ratio displays oscillations as a function of the free precession interval separating the two pulses. Since the frequency of Ramsey fringes corresponds to the value of the detuning of the microwave frequency, in Fig. 4b, the oscillations due to both resonances add constructively, while in Fig. 4c, they display a beating signal between two frequencies with one being twice the other. The decay of the Ramsey fringes gives a direct measurement of the dephasing time of the electronic spin *T*_2_*=115±9 ns at 3.6 K. This value is commensurate with the characteristic time of population transfer between the two orbital branches of the ground state, taken as twice the orbital decay time 2*T*_1,orbital_=133±4 ns at 3.6 K in our measurements. Shorter dephasing times have previously been obtained at slightly higher temperatures through coherent population trapping^19^,^20^, which provides a lower bound due to additional sources of dephasing, including noise from the driving fields. A strain-induced increase of orbital splitting to an energy at which phonon population is larger^27^ is also a cause for shorter dephasing times (see below).

### Single-phonon-mediated spin dephasing and decay

**Figure 5:**
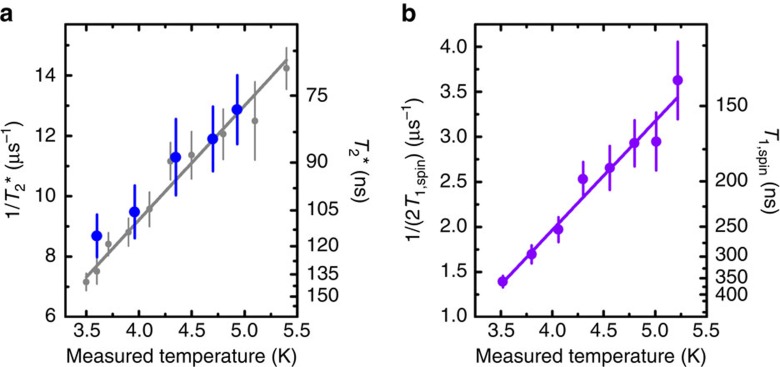
Phonon-induced spin dephasing and decay. (**a**) Evolution of the spin dephasing rate 1/*T*_2_* (blue dots) and orbital population decay rate taken as 1/(2*T*_1,orbital_) (grey dots) as a function of temperature. The solid grey curve is a linear fit of the variation of 1/(2*T*_1,orbital_). (**b**) Temperature dependence of the spin decay rate 1/(2*T*_1,spin_). The solid purple curve is a linear fit of the experimental values. In **a**,**b**, the error bars correspond to the s.e.s. on the fit parameter *T*_2_*, *T*_1,orbital_ and *T*_1,spin_, respectively. In both figures, the *y* axis on the right indicates the coherence times corresponding to the rates of the left axis. The temperature is measured below the sample mount, and, due to heating from the microwave, is expected to be lower than the temperature at the SiV^−^.

The strongest candidate for the cause of limited *T*_2_* is expected to be phonon excitations from the lower to the upper orbital branches in the ground-state manifold. To verify this directly, we measure the spin dephasing rate 1/*T*_2_* as a function of temperature, as shown in Fig. 5a as blue dots. At the temperatures measured in the experiment, the orbital transition rate from the lower to the upper branch of the ground state is expected to be lower than that from the upper to the lower branch by a Boltzmann factor (Supplementary Note 5). Since in our set-up configuration, we underestimate the absolute temperature at the SiV, we take 1/(2*T*_1,orbital_) as a reference for the orbital decay rate and display it as grey dots in Fig. 5a. The orbital decay rate is obtained in the absence of magnetic field similarly to the spin population decay presented in Fig. 1b,c, with initialization into the lower orbital branch of the ground state and measurement of the population recovery into the upper branch (Supplementary Note 6). The orbital decay rate increases linearly with temperature, a signature attributed to single-phonon excitation process between the two orbital branches of the ground state^20^,^28^. The spin dephasing rate follows closely the orbital decay rate dependence on temperature, manifesting that spin dephasing is dominated by single-phonon-mediated transitions between the orbital branches. We also measure the temperature dependence of the spin decay rate 1/(2*T*_1,spin_), as shown in Fig. 5b. Its linear dependence with temperature indicates that it is also dominated by a single-phonon process. The suggested mechanism responsible for spin decay relies on excitation to the upper orbital branch of the ground state through the absorption of a single phonon followed by decay back to the lower branch. Owing to a slight mismatch of spin quantization axes between the two orbitals (Supplementary Note 4), such transitions can result in a spin flip. This mechanism also explains why *T*_1,spin_ can reach several milliseconds^20^ when the magnetic field is aligned with the SiV symmetry axis, which otherwise constitutes a competing quantization axis for the spin via the spin–orbit interaction^19^,^23^. The phonons responsible for those decoherence mechanisms have a frequency around 50 GHz corresponding to the orbital splitting of the SiV^−^ and still have a significant population around 4 K.

## Discussion

In conclusion, we have achieved coherent control of a single SiV^−^ spin with a microwave field, following the identification of spin-flipping transitions through ODMR. We have subsequently implemented Ramsey interferometry to get direct access to the spin dephasing time. The temperature dependence of the dephasing and decay rates reveal that both processes are governed by excitation from the lower to the upper orbital branch of the ground state through single-phonon absorption. This indicates that the coherence times of the SiV^−^ spin will improve significantly by cooling the system to lower temperatures. An alternative approach consists in splitting the orbital branches further apart by applying strain to the SiV^−^, thus increasing the energy required for phonons to cause decoherence. Those approaches would not necessarily improve significantly the coherence time of the orbital-based qubit, which is limited by phonon absorption and emission processes, simultaneously. The latter could be addressed by decreasing the phonon density of states around the frequency of the orbital splitting, which most likely requires nanostructures with dimensions smaller than about 120 nm, corresponding to half the phonon wavelength. Microwave control of the spin offers a strong advantage compared to all-optical control in that it gives much more flexibility for the orientation of the external magnetic field with respect to the SiV^−^ symmetry axis. In particular, control through microwave pulses allows to bring the magnetic field axis close to alignment with the SiV^−^ axis, thus giving access to cycling optical transitions^19^ for single-shot read-out of the spin state^29^. The combination of the optical qualities of the SiV^−^ with the microwave addressing of some of its states also make the SiV^−^ a promising system to be used as a quantum transducer between optical and microwave photons^21^. Finally, the nuclear spin associated with ^29^Si can offer the opportunity of a quantum register, if it can be controlled coherently using radio-frequency pulses and hyperfine coupling to the electron spin.

## Methods

### Sample preparation

The sample used for this experiment is a high-pressure high-temperature type IIa bulk diamond (Element Six) with surface oriented orthogonally to the [111] crystallographic axis. SiV^−^ centres were subsequently created by ion implantation of isotopically purified ^29^Si^+^ ions at an implantation energy of 900 keV. Simulations using the stopping range of ions in matter algorithm^1^ showed that the Si^+^ stop at 500±50 nm below the diamond surface. Implantation doses were tuned from one implantation site to the other from 10^9^ to 10^12^ ions per cm^2^. Following the implantation, the sample was annealed at 1,000 °C in vacuum for 3 h, followed by an oxidation step in air for 1 h at 460 °C. Single SiV^−^ centres have been found in the 10^10^ ions per cm^2^ area.

### Experimental set-up

The diamond sample is mounted in a closed-cycle liquid helium cryostat (Attodry 1000) reading 3.5 K at the sample and the temperature can be tuned via a resistive heater positioned under the sample mount or using heating caused by the microwave pulses. A superconducting coil around the sample space allows to apply a vertical magnetic field from 0 to 9 T. The optical part of the set-up consists of a home-built confocal microscope mounted on top of the cryostat and a microscope objective with numerical aperture=0.82 inside the sample chamber. The sample is moved with respect to the objective utilizing piezoelectric stages (Attocube) on top of which the sample is mounted. SiV^−^ excitation is performed using a frequency tunable diode laser (Toptica DL pro design) whose frequency is maintained in resonance with an optical transition of the SiV^−^ through continuous feedback from a wavemeter (High Finesse WSU). Optical pulses are generated with an acousto-optic modulator (AAOptoelectronic MT350-A0.12-800) controlled by a delay generator (Stanford Research Instruments DG645). The fluorescence from the SiV^−^ is collected through the same objective. The laser light and SiV^−^ fluorescence from the excited transition are filtered out using a home-built monochromator, and the fluorescence from the remaining transitions is sent to an avalanche photodiode (PicoQuant Tau-Spad). The signal from the avalanche photodiode is sent to a time-to-digital converter (qutools quTAU) triggered by the delay generator. The microwave is generated by a frequency generator (Stanford Research Instruments SG384), pulses are generated with a switch connected to the delay generator used for optical pulses. Microwave pulses are then amplified by a microwave amplifier (Mini-Circuits ZHL-16W-43-S+). They travel through semi-rigid cables installed inside the cryostat. A single copper wire (20 μm diameter) positioned on top of the sample allows the microwave to be radiated to <20 μm away from the studied SiV^−^.

### Experimental details

The results presented in Fig. 1b have been acquired with a repetition rate of 500 kHz and an integration time of 300 s. Those in Fig. 2c were obtained with a repetition rate of 500 kHz and an integration time of 200 s per data point. The results in Fig. 3b were recorded with a repetition rate of 20 kHz to minimize heating from the microwave and an integration time of 7,200 s per data point for the green curve and 3,000 s per data point for the orange curve. Finally, the results in Fig. 4b,c were measured with a repetition rate of 50 kHz (to minimize heating) with 2,000 s integration and 200 kHz with 400 s integration, respectively.

### Data availability

The data that support the findings of this study are available from the corresponding author on reasonable request.

## 

## Additional information

**How to cite this article:** Pingault, B *et al*. Coherent control of the silicon-vacancy spin in diamond. *Nat. Commun.* **8**, 15579 doi: 10.1038/ncomms15579 (2017).

**Publisher's note:** Springer Nature remains neutral with regard to jurisdictional claims in published maps and institutional affiliations.

## Supplementary Material

Supplementary Information Supplementary Figures, Supplementary Notes and Supplementary References

Peer Review File
