# Supplementary material for: Coherent control of the silicon-vacancy spin in diamond
Source: Nat Commun. 2017 May 30;8:15579. doi: 10.1038/ncomms15579 (PMC5459988; doi:10.1038/ncomms15579)
Supplement: Supplementary Information — Supplementary Figures, Supplementary Notes and Supplementary References [file ncomms15579-s1.pdf]

Supplementary Note 1. Structure of the SiV^- centre and energy levels

The silicon- vacancy centre consists of two neighbouring carbon atoms replaced by a silicon atom and a vacancy, as shown in Supplementary Fig. 1. The silicon atom lies in the middle of the two lattice sites, with the vacancy being split of each side of the silicon atom. The resulting defect has a D_{3d} symmetry with a main symmetry axis along the <111> directions of the diamond lattice and an inversion symmetry at the silicon atom [1].

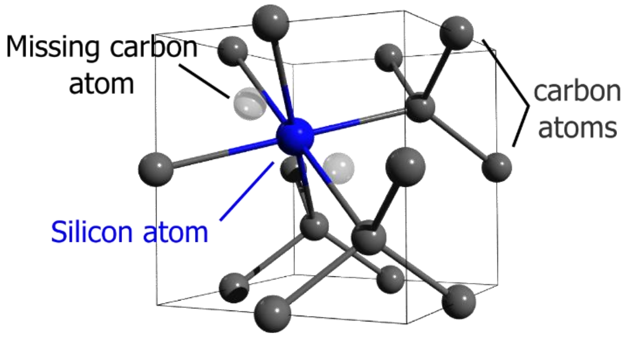

Supplementary Figure 1: Structure of the silicon-vacancy centre in the diamond lattice. Carbon atoms are represented by dark grey spheres, the two missing carbon atoms as light grey spheres and the silicon atom as a blue sphere.

Supplementary Note 2. Enhancement of the photon collection

Diamond is characterised by a high refractive index of n = 2.4. At the interface with vacuum, the large index mismatch results in most photons emitted in the diamond by an SiV^- centre to be reflected, phenomenon known as total internal reflection. In order to circumvent this effect and maximise the photon extraction, hemispherical solid immersion lenses (SIL) have been etched into the sample surface. Photons emitted by a SiV^- located at the centre of a SIL will reach the SIL surface with an angle of incidence of zero which maximises the probability of transmission. Those SILs have been etched using focused ion beam milling (FIB). After the milling, graphite residues and gallium

incorporated into the diamond surface during the FIB process are removed using a post-treatment described in [2].

Each SIL has a radius of 500 nm, corresponding to the average implantation depth of silicon atoms. SILs are organised in arrays randomly located in the area of the sample implanted at a dose corresponding to densities of SiV^- centres allowing isolation of single ones in the laser focus spot. As a consequence, SiV^- centres are randomly located within SILs. The SiV^- studied here is not perfectly located under a SIL. With this emitter, we routinely obtained count rates of approximately 20000 counts/s at saturation in continuous non-resonant excitation and of approximately 500 counts/s at saturation in continuous excitation resonantly with transition D1, which is weakly allowed, as seen in Supplementary Fig. 2. The difference in counts rates between resonant and non-resonant excitation could not be explained but was similar with other SiV^- centres in the same sample.

Supplementary Note 3. Optically detected magnetic resonance signal

The ODMR signal is obtained using 140 ns pulses, heating from which leads to a measured temperature of about 4.3 K. This pulse duration is long enough to limit the broadening of the ODMR peaks while being short enough not to lose contrast due to the relatively short $T_{1,\mathrm{spin}}$ = 200 ns at this temperature. The full width at half maximum of the ODMR peaks is approximately 6.5 ± 0.5 MHz. From the value of T_2^* = 90 ns, one can expect a width of approximately 2 MHz. The relatively short microwave pulse of 140 ns corresponds to a broadening by about 1.1 MHz. The extra width can be attributed to power broadening from the microwave.

The value of 33% for the ODMR contrast is calculated as the ratio between the peak height and the baseline. The ODMR pulse, although longer than the spin dephasing time T_2^* = 90 ns at 4.3 K, remains comparable to it. Hence, the value of the ODMR contrast might differ from the value obtained with much longer microwave pulses (provided $T_{1,\mathrm{spin}}$ is also longer, see below).

Furthermore, the contrast is expected to vary with the initialisation fidelity: A higher initialisation fidelity leads to a stronger decrease in fluorescence by optical pumping, while the fluorescence recovery level due to the resonant microwave pulse remains unchanged, hence a higher overall contrast. In ref. [3], it has been shown that the initialisation fidelity and $T_{1,\mathrm{spin}}$ vary significantly with the magnetic field orientation with respect to the SiV symmetry axis. As a consequence the ODMR contrast as well as the maximal acceptable duration of the microwave pulse vary with the magnetic field orientation.

Supplementary Note 4. Incorporation of the ^{29}Si nuclear spin into the SiV^- Hamiltonian model

The description of the SiV^- centre energy levels has been developed by Hepp et al. in Ref. [4]. This model is based on a group theoretical approach leading to an effective Hamiltonian describing the SiV^- electronic states. This Hamiltonian includes the Jahn-Teller effect, the spin-orbit coupling and the Zeeman effect. The parameters for those effects are deduced by fitting the evolution of the optical transitions predicted by the model as a function of the external magnetic field with the corresponding experimental measurement, as shown in Supplementary Fig. 2. This allows to extract the relative energies of the electronic states of the SiV^- (Supplementary Fig. 3).

This model is here expanded to include interactions due to the ^{29}Si nuclear spin I = 1/2. The hyperfine interaction between the nuclear spin $\mathbf{I}$ and the SiV^- electron spin $\mathbf{S}$ is of the form:

$$\mathcal{H}_{\mathrm{HF}} = \mathbf{I} \cdot \overline{\mathbf{A}} \cdot \mathbf{S} \qquad (1)$$

with $\overline{\mathbf{A}}$ the hyperfine coupling tensor. The hyperfine coupling can be separated into parallel and orthogonal components ($A_{\parallel}$ and $A_{\perp}$, respectively) with respect to the main symmetry axis of the SiV^- centre:

$$\mathcal{H}_{\mathrm{HF}} = A_{\parallel} S_z I_z + A_{\perp}\left(S_x I_x + S_y I_y\right) \tag{2}$$

where S_x, S_y and S_z are the components of the electron spin vector $\mathbf{S}$ and I_x, I_y and I_z are those of the nuclear spin $\mathbf{I}$, with *z* along the SiV^- symmetry axis.

The orbital contribution to the hyperfine coupling is thought to be minor as the measured hyperfine coupling is close to isotropic [4, 5].

We also include the nuclear Zeeman interaction of the form:

$$\mathcal{H}_{\mathrm{nZee}} = -\frac{g_{^{29}\mathrm{Si}}\mu_{\mathrm{n}}}{\hbar}\,\mathbf{I}\cdot\mathbf{B} \tag{3}$$

where μ_{n} is the nuclear magneton, $\hbar$ is the reduced Planck constant, $g_{^{29}\mathrm{Si}}$ is the nuclear Landé factor for ^{29}Si and $\mathbf{B}$ is the applied magnetic field.

From the fit of the energy differences of the resulting hyperfine levels and the measured ODMR spectra, we can extract a value for $A_{\parallel} = 70 \pm 2$ MHz, in agreement with the previously reported values [5] as well as theoretical predictions [6, 7]. At the low magnetic fields studied here (approximately 0.2 T), the quantisation axis for the electron and nuclear spins remains dictated by the spin-orbit coupling [4] and is aligned with the symmetry axis of the SiV^-. Thus, $A_{\perp}$ does not influence the measured hyperfine splitting in this configuration.

The hyperfine level splitting results from an interplay between the hyperfine interaction, the electronic spin orbit coupling and the orientation and magnitude of the external magnetic field. In our experiment, the angle between the magnetic field and the symmetry axis of the SiV^- centre is approximately 109°, which leads to a level splitting of approximately 27 MHz, hence a 54 MHz splitting between the measured ODMR peaks. In ref. [3], the angle between the applied magnetic field and the SiV axis for the $^{29}SiV^-$ centre studied is not mentioned. Assuming an angle of 70° as for the ^{28}SiV measurement mentioned earlier in the text, we obtain a level splitting of approximately 35 MHz, consistent with the 69 MHz splitting between the two CPT dips observed experimentally.

From the model, we deduce the tomography of the ground state manifold (Supplementary Fig. 4 and 5). Each state is dominated by a given projection of the electron spin and of the nuclear spin, but also includes weaker components of the other projections, especially for the electron spin. Furthermore, those weaker components differ between states of the lower orbital branch and those of the upper orbital branch. This can be interpreted as slightly different effective quantisation axes for the electron spin between the two branches.

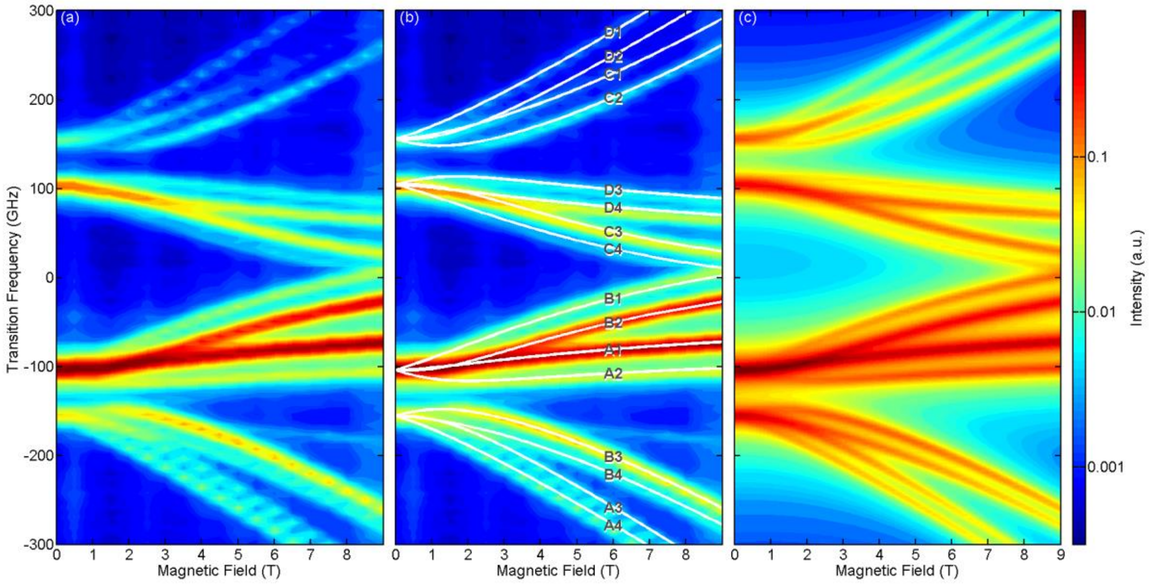

Supplementary Figure 2: Zeeman spectrum of the SiV$^-$ centre studied. The magnetic field is applied along the [-1-11]-direction. (a) Experimental spectra alone, (b) same spectra overlapped with simulated transition wavelengths (white lines) and the corresponding transition labels. (c) Fully simulated spectra as a function of magnetic field.

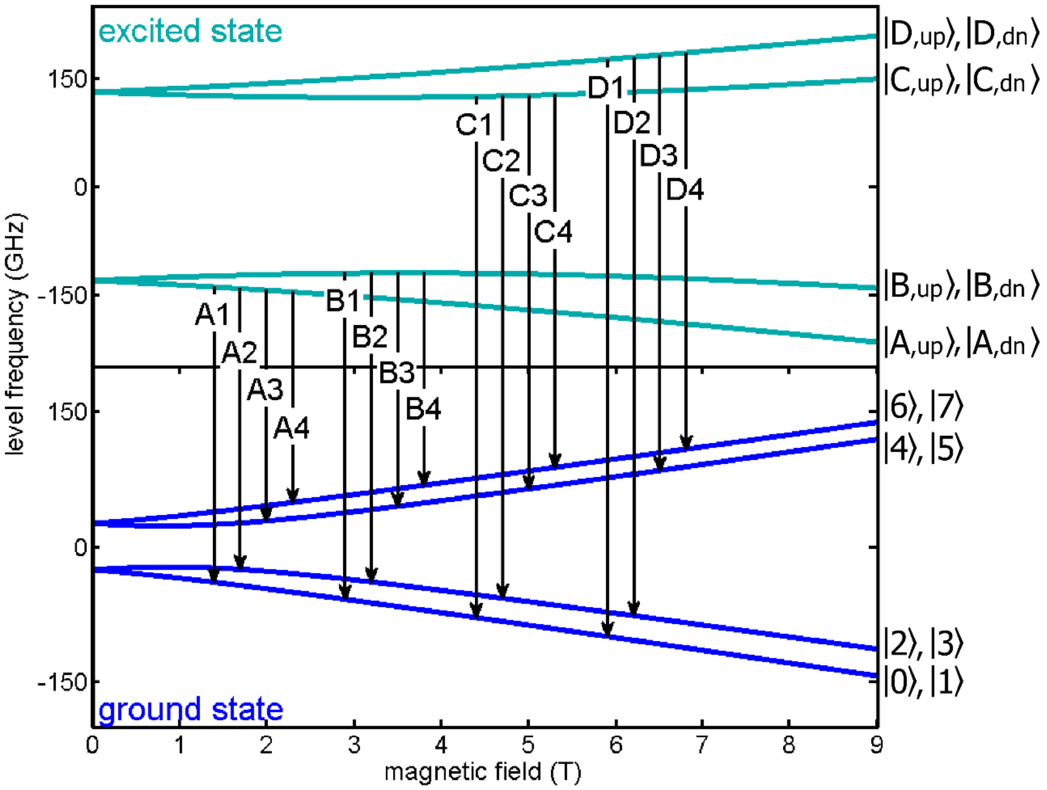

Supplementary Figure 3: Simulated energy levels of the SiV^- centre. The energy levels correspond to the simulated transitions of Supplementary Fig. 2. The hyperfine splitting in both ground and excited states is too small to be visible at that scale. In the ground state, hyperfine-split levels are labelled from 0 to 7. In the excited state, the labelling follows that introduced in Ref. [4], with each label referring to the electronic state from A to D and the nuclear spin state up or down.

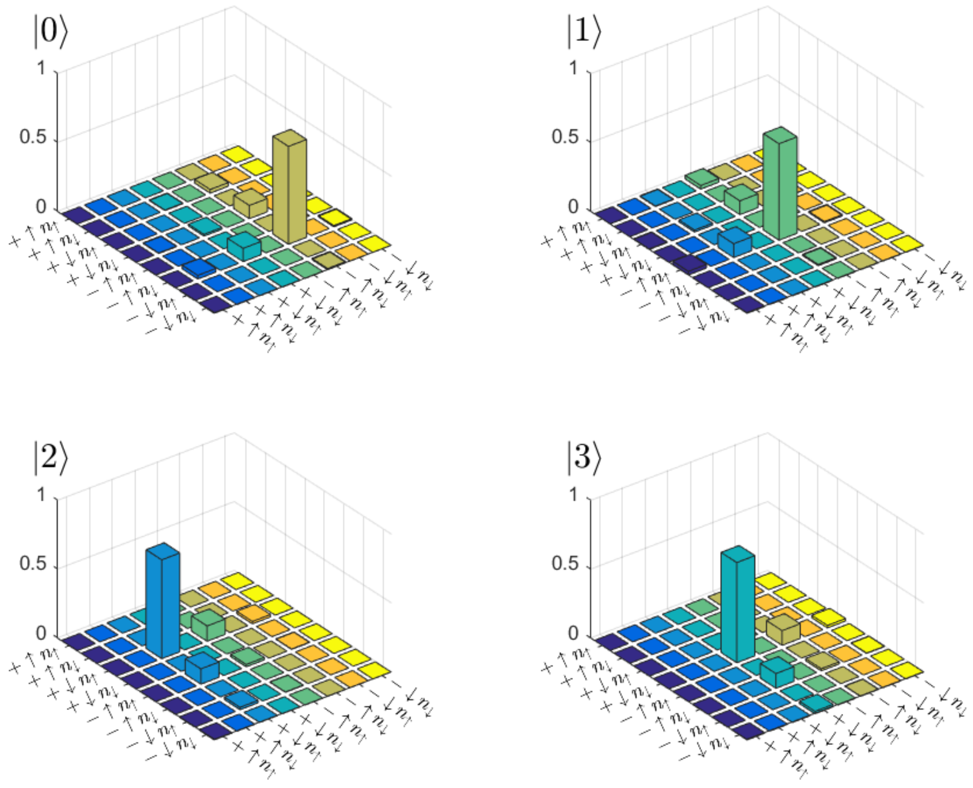

Supplementary Figure 4: Tomography of the four states in the lower ground state orbital. The magnetic field is at *B* = 0.214 T. The state labels are as in Supplementary Fig. 2. The tomography is plotted as the square of the norm of the density matrix elements. Basis states correspond to the eigenstates of the orbital operator L_z, the electronic spin operator S_z and the nuclear spin operator I_z [4].

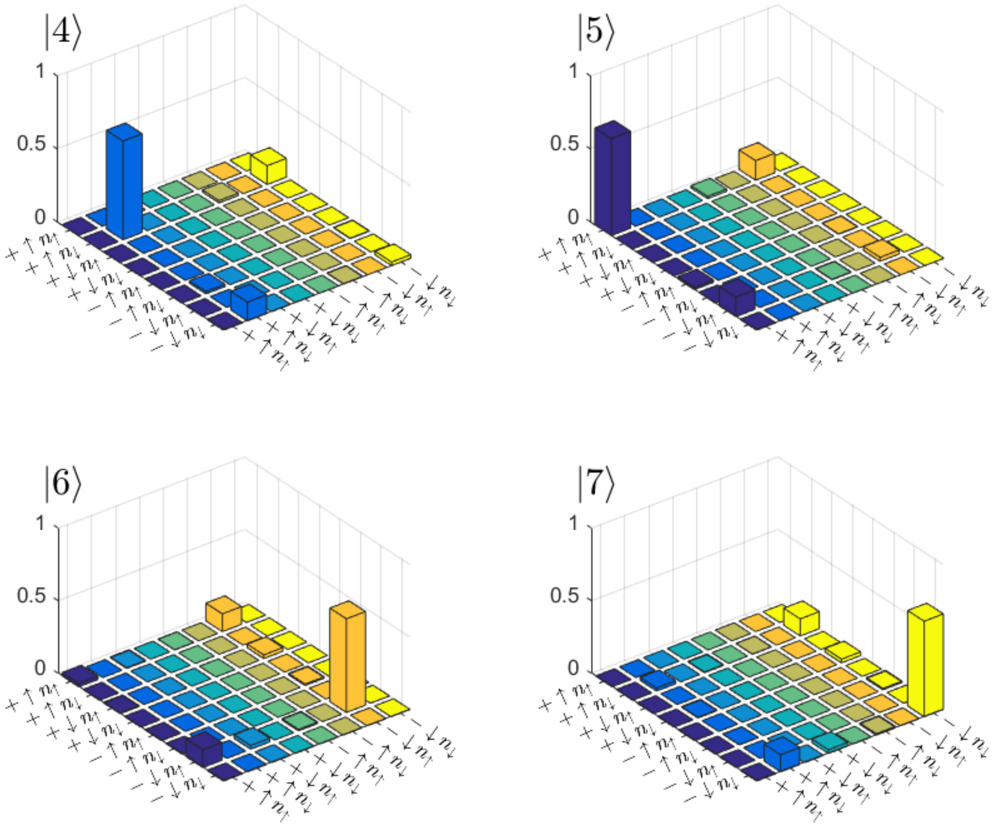

Supplementary Figure 5: Tomography of the four states in the upper ground state orbital. The magnetic field is at B = 0.214 T. The state labels are as in Supplementary Fig. 2. The tomography is plotted as the square of the norm of the density matrix elements. Basis states correspond to the eigenstates of the orbital operator L_z, the electronic spin operator S_z and the nuclear spin operator I_z [4].

Supplementary Note 5. Master equation model for Rabi oscillations

The dynamics of the SiV⁻ in the presence of a microwave pulse is modelled using an eight-level master equation in Lindblad form [8].

The eight levels considered correspond to the two branches of the ground state. Each branch is split into two by the Zeeman interaction and the spin-orbit coupling acting on the electronic spin. Each level is further split by the hyperfine interaction. This results in the energy levels depicted in Supplementary Fig. 6. Energy states are thus characterised by the projections of the electron and nuclear spins. As in Supplementary Fig. 3, they are labelled from 0 to 7.

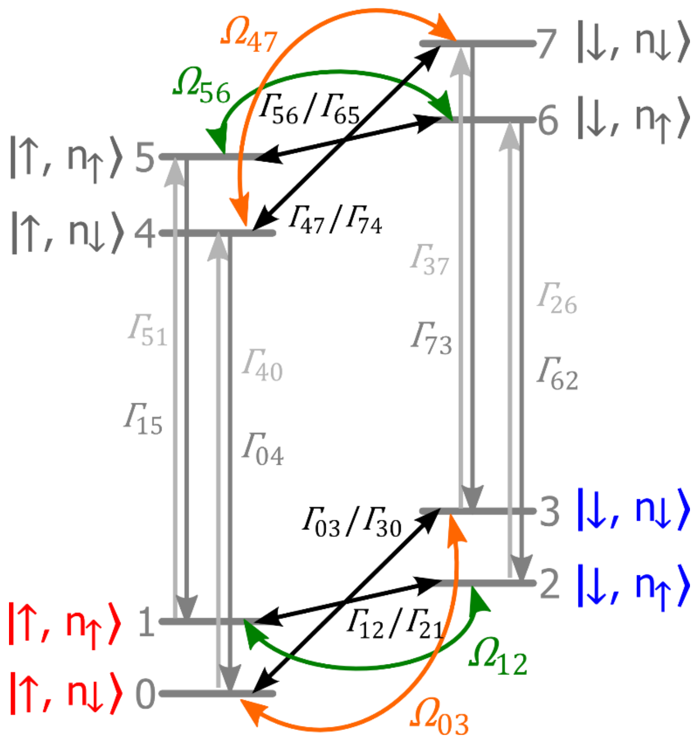

Supplementary Figure 6: Representation of the energy levels of the ground state of the SiV⁻. The levels in the lower branch are labelled from 0 to 3 and those in the upper branch are labelled from 4

to 7. Arrows indicate the different decay rates Γ_{ij} and Rabi frequencies Ω_{ij} between levels *i* and *j* considered in the master equation model.

The master equation is of the form:

$$\frac{\mathrm{d}\rho}{\mathrm{d}t} = \frac{\mathrm{i}}{\hbar}[\rho, \mathcal{H}] + \mathcal{L}(\rho) \tag{4}$$

where ρ is the density matrix for the SiV^- states considered, $\hbar$ is the reduced Planck constant, $\mathcal{H}$ is the Hamiltonian of the system formed by the SiV^- levels considered and the microwave field in the rotating wave approximation, $\mathcal{L}(\rho)$ is the Lindblad superoperator.

The Hamiltonian of the system in the rotating wave approximation can be written as follows:

$$\mathcal{H} = \begin{pmatrix} E_0+\omega & 0 & 0 & \Omega_{03}/2 & 0 & 0 & 0 & 0 \\ 0 & E_1+\omega & \Omega_{12}/2 & 0 & 0 & 0 & 0 & 0 \\ 0 & \Omega_{12}/2 & E_2 & 0 & 0 & 0 & 0 & 0 \\ \Omega_{03}/2 & 0 & 0 & E_3 & 0 & 0 & 0 & 0 \\ 0 & 0 & 0 & 0 & E_4+\omega & 0 & 0 & \Omega_{47}/2 \\ 0 & 0 & 0 & 0 & 0 & E_5+\omega & \Omega_{56}/2 & 0 \\ 0 & 0 & 0 & 0 & 0 & \Omega_{56}/2 & E_6 & 0 \\ 0 & 0 & 0 & 0 & \Omega_{47}/2 & 0 & 0 & E_7 \end{pmatrix} \tag{5}$$

where E_i are the energies of the 8 levels considered, Ω_{ij} are the Rabi frequencies between levels *i* and *j*, and ω is the frequency of the microwave. The state labelled 0 is taken as the origin of the energy scale. The energies of the other levels of the lower branch of the ground state are deduced from the ODMR spectrum at an applied magnetic field of 0.214 T:

$$E_0 = 0 \text{ GHz} \tag{6}$$
$$E_1 = 2\pi \cdot 0.027 \text{ GHz} \tag{7}$$
$$E_2 = 2\pi \cdot 2.899 \text{ GHz} \tag{8}$$
$$E_3 = 2\pi \cdot 2.926 \text{ GHz} \tag{9}$$

The uncertainties on those values are 0.2 MHz from the Lorentzian fits of the ODMR peaks. The energies of the levels in the upper branch are extracted from the expanded group theoretical model described in Supplementary Note 4 (with uncertainties of about 10 MHz through calibration of the model parameters with experimental values of the optical and ODMR transition energies):

$$E_4 = 2\pi \cdot 51.61 \text{ GHz} \tag{10}$$

$$E_5 = 2\pi \cdot 51.63 \text{ GHz} \tag{11}$$
$$E_6 = 2\pi \cdot 54.24 \text{ GHz} \tag{12}$$
$$E_7 = 2\pi \cdot 54.26 \text{ GHz} \tag{13}$$

The microwave-induced bare Rabi frequencies are extracted from the Rabi oscillations curves fitted by a sine function with an exponential decay:

$$\Omega_{12} = 2\pi \cdot 14.7 \text{ MHz} \tag{14}$$
$$\Omega_{03} = 2\pi \cdot 14.4 \text{ MHz} \tag{15}$$

The bare Rabi frequencies in the upper branch are assumed to be similar with $\Omega_{47} = \Omega_{56} = 2\pi \cdot 14.5 \text{ MHz}$.

The Lindblad superoperator, accounting for the system relaxation due to the coupling of the SiV^- to its environment, is of the form:

$$\mathcal{L}(\rho) = \sum_{i,j} \mathcal{L}_{ij}(\rho) = -\frac{1}{2}\sum_{i,j}\left(C_{ij}^{\dagger}C_{ij}\rho + \rho C_{ij}^{\dagger}C_{ij}\right) + \sum_{i,j} C_{ij}\rho\, C_{ij}^{\dagger} \tag{16}$$

with the collapse operators $C_{ij} = \sqrt{\Gamma_{ij}}|j\rangle\langle i|$ describing the relaxation from state *i* to state *j* with decay rate Γ_{ij}.

The electronic spin decay rates within the lower branch are directly linked to the spin population decay time $T_{1,\text{spin}}$ through:

$$\Gamma = \frac{1}{2T_{1,\text{spin}}} \tag{17}$$

All the rates are assumed to be equal, thus giving:

$$\Gamma_{03} = \Gamma_{30} = \Gamma_{12} = \Gamma_{21} = 1.39 \text{ MHz} \tag{18}$$

The corresponding decays within the upper branch are assumed equal to those of the lower branch, giving:

$$\Gamma_{47} = \Gamma_{74} = \Gamma_{56} = \Gamma_{65} = 1.39 \text{ MHz} \tag{19}$$

Experimentally, the microwave causes heating of the sample, the temperature varying linearly with the microwave pulse duration. This implies that the sample temperature is slightly elevated as the microwave pulse duration increases. A measurement of $T_{1,\mathrm{spin}}$ as a function of the temperature T due to the microwave and measured below the sample mount has been realised and $1/(2T_{1,\mathrm{spin}})$ varies linearly with *T*, as seen in Fig. 5b of the main text. The linear fit (purple curve) gives a dependence $1/(2T_{1,\mathrm{spin}})(\mathrm{MHz}) = (1.2 \pm 0.1)(\mathrm{MHz.K^{-1}}) * T(\mathrm{K}) - (2.9 \pm 0.4)(\mathrm{MHz})$. The uncertainties correspond to the standard deviations of the fit for the associated coefficients. Units are indicated between parentheses.

Such a variation is accounted for in the model and the value for $T_{1,\mathrm{spin}}$ is changed according to the temperature measured experimentally for a given duration of the microwave pulse. It should be noted that due to the way the sample is mounted, the measured temperature is expected to be lower than the temperature at the sample.

The spin-preserving interbranch downward and upward rates (Γ_{down} and Γ_{up} respectively) between the ground state orbital branches, involving respectively the creation or annihilation of a phonon, are directly linked to the orbital population decay time $T_{1,\mathrm{orbital}}$ as:

$$\frac{1}{T_{1,\mathrm{orbital}}} = \Gamma_{\mathrm{up}} + \Gamma_{\mathrm{down}} \tag{20}$$

With $\Gamma_{\mathrm{up}} = \Gamma_{\mathrm{down}} \cdot e^{-\Delta E/k_{\mathrm{B}}T}$ where k_{B} is the Boltzmann constant and ΔE is the energy difference between the lower and upper branches, taken to be approximately 52.4 GHz. This gives the downward rates:

$$\Gamma_{51} = \Gamma_{40} = \Gamma_{73} = \Gamma_{62} = \frac{1}{T_{1,\mathrm{orbital}}\left(1 + e^{-\Delta E/k_{\mathrm{B}}T}\right)} \tag{21}$$

And the upward rates:

$$\Gamma_{15} = \Gamma_{04} = \Gamma_{37} = \Gamma_{26} = \frac{e^{-\Delta E/k_{\mathrm{B}}T}}{T_{1,\mathrm{orbital}}\left(1 + e^{-\Delta E/k_{\mathrm{B}}T}\right)} \tag{22}$$

This guarantees a Boltzmann population between the lower and upper branches.

The microwave-induced heating of the sample is also taken into account for those rates, as $1/(2T_{1,\mathrm{orbital}})$ increases linearly with the temperature (see Fig. 5a of the main text) [3, 9]. The linear fit (grey curve) gives a dependence $1/(2T_{1,\mathrm{orbital}})(\mathrm{MHz}) = (3.8 \pm 0.2)(\mathrm{MHz.K^{-1}}) * T(\mathrm{K}) - (5.9 \pm 0.8)(\mathrm{MHz})$. The uncertainties correspond to the standard deviations of the fit for the associated coefficients. Units are indicated between parentheses.

The electron spin-flipping interbranch rates as well as all nuclear spin-flipping rates are considered negligible compared to the previously mentioned rates.

The initial populations of the different states are taken as follows:

$$\rho_{00} = \rho_{11} = 0.5 * (1 - \mathbb{i}) * \frac{1}{1 + e^{-\Delta E/k_{\mathrm{B}}T}} \tag{23}$$

$$\rho_{22} = \rho_{33} = 0.5 * \mathbb{i} * \frac{1}{1 + e^{-\Delta E/k_{\mathrm{B}}T}} \tag{24}$$

$$\rho_{44} = \rho_{55} = 0.5 * (1 - \mathbb{i}) * \frac{e^{-\Delta E/k_{\mathrm{B}}T}}{1 + e^{-\Delta E/k_{\mathrm{B}}T}} \tag{25}$$

$$\rho_{66} = \rho_{77} = 0.5 * \mathbb{i} * \frac{e^{-\Delta E/k_{\mathrm{B}}T}}{1 + e^{-\Delta E/k_{\mathrm{B}}T}} \tag{26}$$

where a Boltzmann factor is assumed to account for the population difference between upper and lower orbital branches, a factor 0.5 arises from the assumption that states differing only by their nuclear spin states (0 and 1, 2 and 3, 4 and 5, 6 and 7) are populated equally, and $\mathbb{i}$ is the value of the initialisation. This initialisation value is taken as an adjustable parameter. The best fit to data is obtained for a value of 85%.

The master equation is solved during a time interval corresponding to the experimental delay of 210 ns between the end of the optical initialisation pulse and the optical readout pulse. The same procedure is repeated for a microwave pulse duration ranging from 0 to 200 ns, as realised experimentally. An example of the evolution of the different states populations in time for a microwave duration of 100 ns is given in Supplementary Fig. 7. For each of these curves, the final population at 210 ns is plotted as a function of the microwave duration, as shown in Supplementary Fig. 8. The simulated Rabi curve displayed in Fig. 3b of the main text corresponds to the sum of the populations of level 0 and 1, which are addressed by the resonant laser pulse, with a scaling factor.

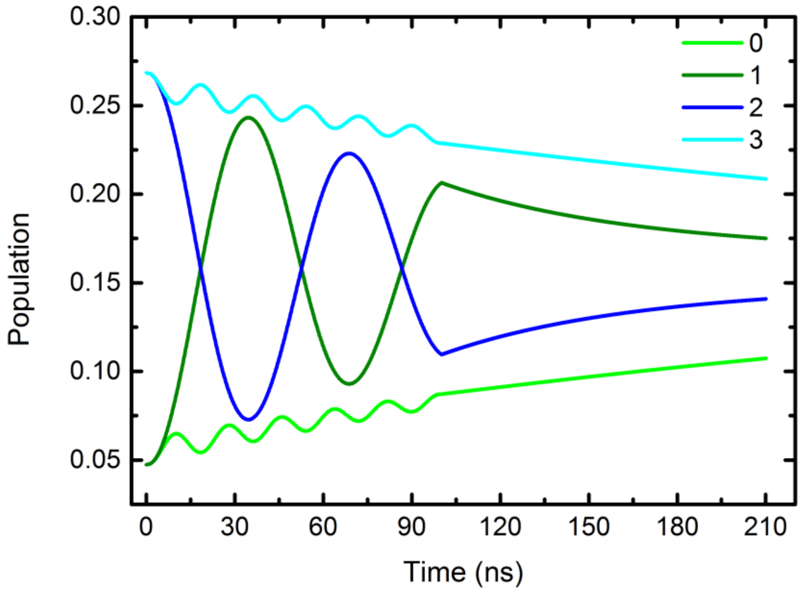

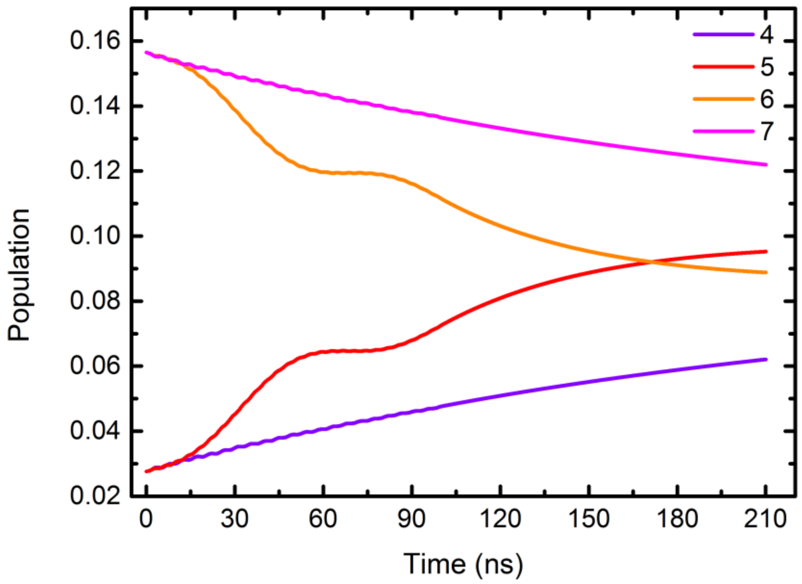

Supplementary Figure 7: Simulated evolution of the populations in the ground state levels. The levels in the lower branch are labelled from 0 to 3 and those in the upper branch, from 4 to 7. The microwave pulse is 100 ns long and in resonance with the transition between levels 1 and 2.

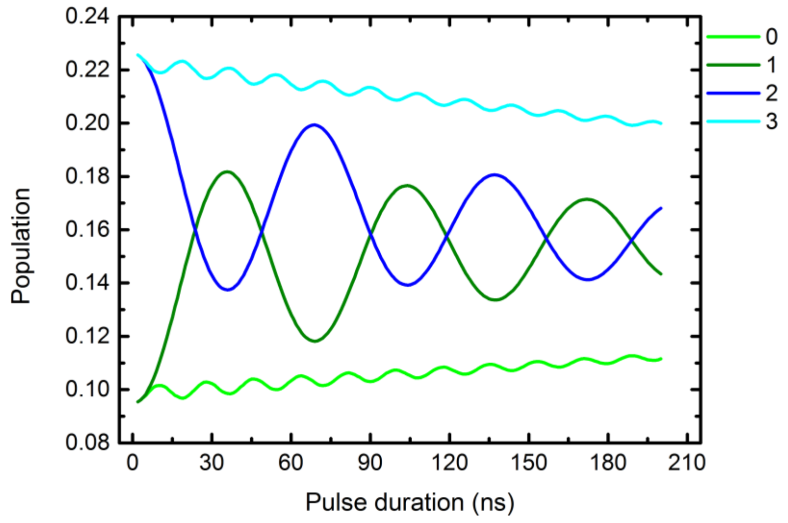

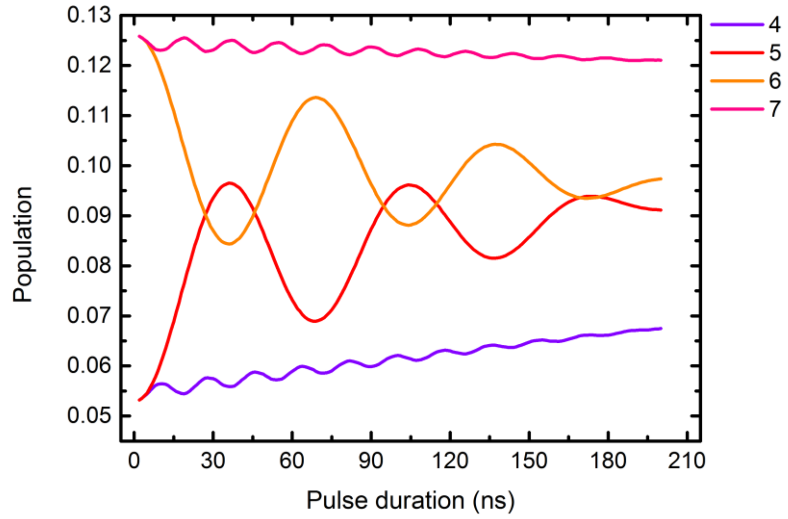

Supplementary Figure 8: Simulated evolution of the final population in the ground state levels.

The final populations correspond to those after 210 ns. The final populations are plotted as a function of the duration of a microwave pulse resonant with the transition between levels 1 and 2.

Supplementary Note 6. Measurement of orbital population decay time

The orbital population decay time $T_{1,\mathrm{orbital}}$ from the lower ground state orbital branch to the upper ground state orbital branch is measured in the absence of external magnetic field through a pump-probe experiment similar to that described in the main text for Fig. 1b and c. In the absence of magnetic field, the Zeeman sublevels in the ground state (1 - 2 and 3 - 4), as well as those in the excited state (A – B and C - D) are degenerate. A first 500 ns laser pulse resonant with the transition from the upper ground state orbital branch (3 - 4) to the upper excited state orbital branch (C - D) optically pumps the SiV^- into the lower ground state orbital branch (1 - 2). After a waiting time Δt, a second 500 ns pulse identical to the first one reads out the population recovery into the upper ground state orbital branch (3 - 4). By measuring the recovery through the peak ratio (as described in the main text) as a function of the delay time, an exponential fit allows to extract the value of $T_{1,\mathrm{orbital}}$. This measurement is performed at several measured temperatures, an example of which is shown in Supplementary Fig. 9.

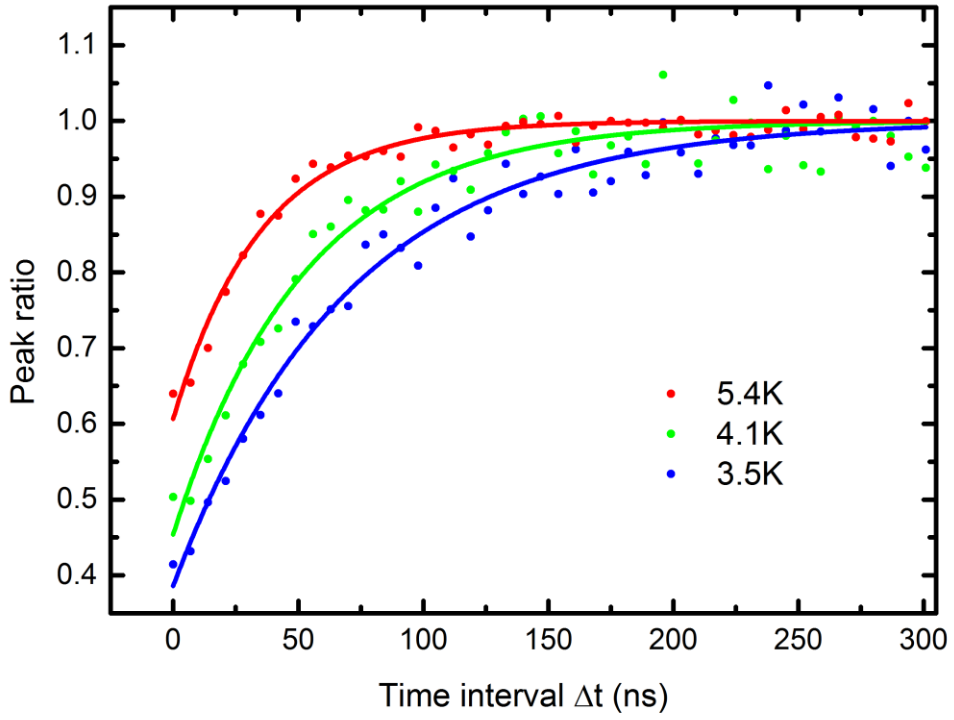

Supplementary Figure 9: Orbital population decay time at different temperatures. The decay corresponds to that from the lower ground state orbital to the upper one. The measured temperature values are indicated in legend. The curves correspond to exponential fits from which $T_{1,\mathrm{orbital}}$ is extracted as the $1/e$ value.

Supplementary References

[1] Goss, J.P., *et al.* The Twelve-Line 1.682 eV Luminescence Center in Diamond and the Vacancy-Silicon Complex. *Phys. Rev. Lett.* **77**, 3041-3044 (1996).
[2] Riedrich-Möller, J. One- and two-dimensional photonic crystal microcavities in single crystal diamond. *et al*., *Nat. Nano.* **7**, 69-74 (2012).
[3] Rogers, L. J. *et al*., All-Optical Initialization, Readout, and Coherent Preparation of Single Silicon-Vacancy Spins in Diamond. *Phys. Rev. Lett*. **113**, 263602 (2014).
[4] Hepp, C. *et al*., Electronic Structure of the Silicon Vacancy Color Center in Diamond. *Phys. Rev. Lett.* **112**, 036405 (2014).
[5] A.M. Edmonds, et al., Electron paramagnetic resonance studies of silicon-related defects in diamond. *Phys. Rev. B* **77**, 245205 (2008).
[6] Goss, J. P. *et al.* Density functional simulations of silicon-containing point defects in diamond. *Phys. Rev. B* **76**, 075204 (2007).
[7] Gali, A. and Maze, J. R. Ab initio study of the split silicon-vacancy defect in diamond: Electronic structure and related properties. *Phys. Rev. B* **88**, 235205 (2013).
[8] Carmichael, H.J. Statistical Methods in Quantum Optics 1, Springer (1999).
[9] Jahnke, K. D. *et al*., Electron–phonon processes of the silicon-vacancy centre in diamond. *New J. Phys*. **17**, 043011 (2015).
